# Isolation and purification of antibacterial compound from *Streptomyces levis* collected from soil sample of north India

**DOI:** 10.1371/journal.pone.0200500

**Published:** 2018-07-10

**Authors:** Vineeta Singh, Shafiul Haque, Shruti Khare, Anil Kumar Tiwari, Diksha Katiyar, Bikram Banerjee, Krishna Kumari, C. K. M. Tripathi

**Affiliations:** 1 Microbiology Division, CSIR - Central Drug Research Institute, Lucknow, Uttar Pradesh, India; 2 Department of Biotechnology, Institute of Engineering and Technology, Lucknow, Uttar Pradesh, India; 3 Research and Scientific Studies Unit, College of Nursing and Allied Health Sciences, Jazan University, Jazan, Saudi Arabia; 4 Department of Chemistry, Lucknow University, Lucknow, Uttar Pradesh, India; 5 Department of Chemistry, MMV, Banaras Hindu University, Varanasi, Uttar Pradesh, India; 6 Department of Biotechnology, Shri Ramswaroop Memorial University, Lucknow, Uttar Pradesh, India; Tallinn University of Technology, ESTONIA

## Abstract

During the screening programme for microbial cultures producing antimicrobial agents, an active microbial strain of *Streptomyces* was isolated from the agricultural soil of Narnaul, Haryana India. Physiological, biochemical characteristics and 16S ribosomal RNA sequence homology studies revealed that it was similar to *Streptomyces levis* (sequence similarity 100%). The microbial strain was submitted to Genomebio Technologies Pvt. Ltd., Pune, Maharashtra, India under Accession No. EU124569. The isolated strain was found to produce extracellular active compound showing strong antimicrobial activity against *Klebsiella pneumoniae* MTCC 109, *Pseudomonas aeruginosa* MTCC 741 and *Staphylococcus aureus* MTCC 96. The antibacterial compound was successfully isolated and purified. Structure elucidation of antibacterial metabolite with EI-MS/ HRMS showed molecular ion peak at m/z 686 [M+H]^+^. Whereas, elemental analysis of the said compound showed C = 61.31, H = 8.61, N = 2.04 and O = 28.02, and indicated a molecular formula of C_35_H_59_NO_12_. The presence of ‘chromone’ nucleus in the compound’s chemical structure was confirmed by using ^1^HNMR studies. The present study reports the purification of potential antibacterial compound from *Streptomyces levis* isolated from the unexplored soil of north India and warrants for further characterization of this potential compound for optimum utilization for antimicrobial purposes.

## Introduction

During the past two decades, among gram negative bacteria group *Klebsiella pneumonia* and *Pseudomonas aeruginosa* has emerged as an important pathogen that can cause severe diseases in animals, including humans. *K*. *pneumoniae* is known to cause destructive changes to human lungs via inflammation and hemorrhage with cell death (necrosis) that sometimes produces a thick, bloody, mucoid sputum [1]. Whereas, *P*. *aeruginosa* typically infects the pulmonary tract, urinary tract, burns, wounds, and also causes other blood infections [2]. In addition, with the emergence of resistance against commercially available antibiotics, an urgent need of new compounds has been increased. Recently, Nekidy, et al., [3] describe the use of combination of ertapenem/meropenem with minimum inhibitory concentration (MIC) values for the treatment of various infections caused by multi-drug resistant *Klebsiella pneumonia*. In a case of immuno-compromised patients, the use of high doses of drugs again increases the risk of side effects and creates the need of wide-spectrum potential drugs.

Natural products are the most consistent auspicious source of biologically active molecules of greater chemical/structural diversity and offer opportunities for finding novel drug lead compounds that are active against wide range of targets [4–6]. The main producers of the secondary metabolites are the microbes and they are continued to be useful source of novel secondary metabolites with a range of biological activities [7]. Among microbes, most of the known antibiotics are isolated from actinomycetes group of bacteria and ~75% of commercially useful antibiotics are produced by different *Streptomyces* species [8,9]. Therefore, screening, isolation and characterization of promising strains of actinomycetes capable of producing potential antibiotics has been a major area of research for many years [10].

In the present study, isolation of *Streptomyces levis* producing antimicrobial metabolite having potent activity against *Klebsiella pneumonia* and *Pseudomonas aeruginosa* has been explained. Additionally, production, purification, structure elucidation and bioactivity of the isolated antimicrobial metabolite have also been discussed.

## Materials and methods

### Isolation and fermentation of the strain showing antimicrobial activity

Antimicrobial metabolite producing actinomycetes strain was isolated from the soil sample collected from a hospital surrounding of Narnaul (Haryana). Stock culture was maintained on M6 slants (glucose 4 g, yeast extract powder 4 g, malt extract powder 10 g, CaCO_3_ 2 g, agar powder 2%, distilled water 1L, pH 6.8). Morphological, biochemical, cultural and physiological tests were performed according to the methods described by Holt *et al*. [11]. Species level characterization of the microbial strain was commercially performed at Genomebio Technologies Pvt. Ltd., Pune, Maharashtra, India.

For the production of antimicrobial metabolite, the isolated culture was inoculated into 250 mL flasks containing 50 mL of X-medium (soyabean meal 10 g, CaCO_3_ 3 g, MgSO_4_.7H_2_O 0.5 g, (NH_4_)_2_HPO_4_ 0.5 g, NaCl 3 g, K_2_HPO_4_ 1 g, glycerol 15 mL, DW 1L, pH 6.9–7.0) as seed and incubated at 28°C and 200 rpm on a rotary shaker for 48 h, which was used as seed culture. 0.5% of this seed was transferred to 1L flask containing 200 mL of X-medium as production flasks and incubated at 28°C and 200 rpm on a rotary shaker for 96 h. Antimicrobial activity of the isolated strain was checked by agar well method using 100 μl of the fermented broth against *Staphylococcus aureus* MTCC 96, *Pseudomonas aeruginosa* MTCC 741, and *Klebsiella pneumonia*e MTCC 109.

Further, the activity of the isolated ‘active’ compound was quantitatively determined in terms of minimum inhibitory concentration (MIC) employing broth dilution method using 200 μg/ml as the starting concentration of the compound. MICs of the isolated active compound were studied with the serial dilution method as recommended by NCCLS (formerly known as the National Committee for Clinical Laboratory Standards) guidelines [12]. All chemicals and reagents were procured from Sigma Aldrich, Lancaster, Merck, SD Fine-Chem Ltd, and Spectrochem Pvt. Ltd, and were used without purification.

### Purification of the antimicrobial metabolite

Ninety-six h old fermented culture was filtered using Buchner funnel to separate the biomass from the broth. The cell free broth thus obtained was extracted twice with ethyl acetate. The organic phase was concentrated to dryness under vacuum using a rotary evaporator (Buchi labs, Switzerland). The activity of the concentrated crude extract against the pathogenic strain was monitored by the agar diffusion paper-disc method.

The purification of antimicrobial metabolite was carried out using normal phase silica gel (mesh size 230–400) column (1.8 X 70 cm) as a packing material. Loading slurry was made by mixing 1gm silica gel with 0.5 gm antimicrobial crude in methanol and dried on rotary evaporator. The column was eluted with stepwise methanol—chloroform gradient solvent system (0.0:100%). Eluted solvent fractions were collected and dried on rotary evaporator at 37°C. All the fractions were checked for antimicrobial activity against *S*. *aureus* MTCC 96, *K*. *pneumoniae* MTCC 109 and *P*. *aeruginosa* MTCC 741 by disc diffusion method. The purity of the active fractions was tested by using HPLC system (Waters), equipped with 515 HPLC binary pump, 2998 Photodiode array (PDA) detector, and 2707 autosampler. HPLC separation was achieved on a reverse phase, reverse phase silica column (RP18) with a mobile phase consisting of water—acetonitrile gradient at the flow rate of 1 ml/min.

### Chemical characterization of the purified compound

IR Spectrum (potassium bromide) was recorded on Perkin-Elmer FTIR spectrophotometer (ν_max_ in cm^-1^). ESI mass spectrum was recorded on a V_a_ 70-70H mass spectrometer (Manchester, UK) at 70 eV with a trap current of 200 μA and 4 kV of acceleration voltage and ESI mode positive ion trap detector. Elemental analysis was performed on a Perkin-Elmer 2400 series II elemental CHNS analyzer. ^1^H- and ^13^C-NMR spectrum were recorded on Bruker 200/300 MHZ instruments using CDCl_3_ and DMSO as solvents. Chemical shifts (δ) were reported in ppm downfield using internal tetramethylsilane (TMS) standard.

## Results

### Antimicrobial activity

During the screening process, six isolates were found to have significant antimicrobial activity against *K*. *pneumoniae* MTCC 109. One of the isolate (RS 25) showed significant antimicrobial activity against gram-positive *S*. *aureus* MTCC 96 as well as gram-negative *P*. *aeruginosa* MTCC 741 and *K*. *pneumoniae* MTCC 109 with the zone of inhibition of 24, 20 and 23 mm, respectively, when checked by using agar well method. The MIC values against the above stated test strains were found to be 6.25, 12.5 and 6.25 μg/ml, respectively.

### Cultural and physiological characterization

**Fig 1:**
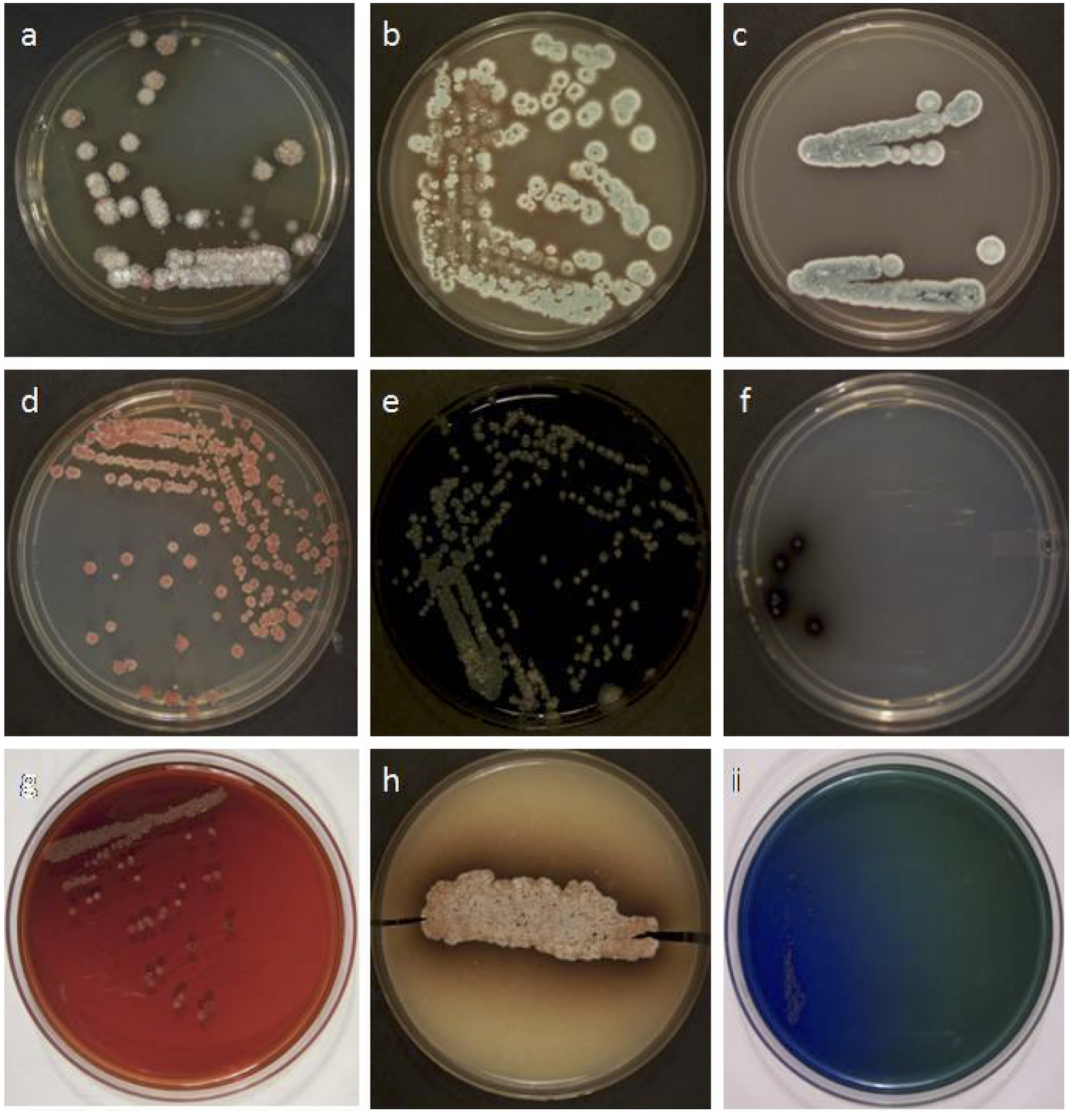
Growth of *S*. *levis* on different growth medium, a: ISP-2, b: ISP-3, c: ISP-4, d: ISP-5, e: ISP-6, f: ISP-7, g: Mac Conkey Agar, h: Casein Agar, i: Citrate utilization.

**Fig 2:**
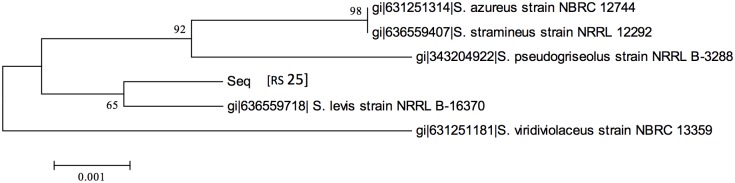
Phylogenetic tree showing evolutionary relationship of *Streptomyces levis* with other members of *Streptomyces* along with their evolutionary distances.

The producer strain (RS-25) was characterized by using morphological and cultural characteristics like growth intensity, colony morphology and formation of soluble pigments on various International Streptomyces Project (ISP) media. The results of cultural and physiological characteristics are summarized in Table 1. The bacterial culture was able to utilize various complex (casein) and simple media (amylase) for the growth. The strain was found to produce certain extracellular enzymes like protease, amylase, nitrate reductase, urease and asparaginase as verified by the growth on a variety of ISP media except tyrosine agar (ISP7) (Fig 1). Finally, RS-25 was characterized on the basis of 16S rRNA homology sequence studies at Genomebio Technologies Pvt. Ltd., Pune, Maharashtra, India and identified as *Streptomyces levis* under Accession No. EU124569. A total of 1152 nucleotide bases of the strain were used to generated the phylogentic tree (Fig 2).

**Table 1:** Observation table of morphological characteristics on different ISP medium.

S. No.	ISP Medium	Growth	Colony morphology	Pigmentation
1	ISP-2	Moderate	Circular; Convex and Pale white colonies	Pink
2	ISP-3	Abundant	Irregular; Slightly Convex and light pink colonies	Red
3	ISP-4	Moderate	Circular; Flat and Light green colonies	Red
4	ISP-5	Abundant	Circular; Convex and red colonies	Red
5	ISP-6	Abundant	Circular; Convex	Black
6	ISP-7	Slight	Irregular; Raised and blackish colonies	Black

### Purification of the active compound

**Fig 3:**
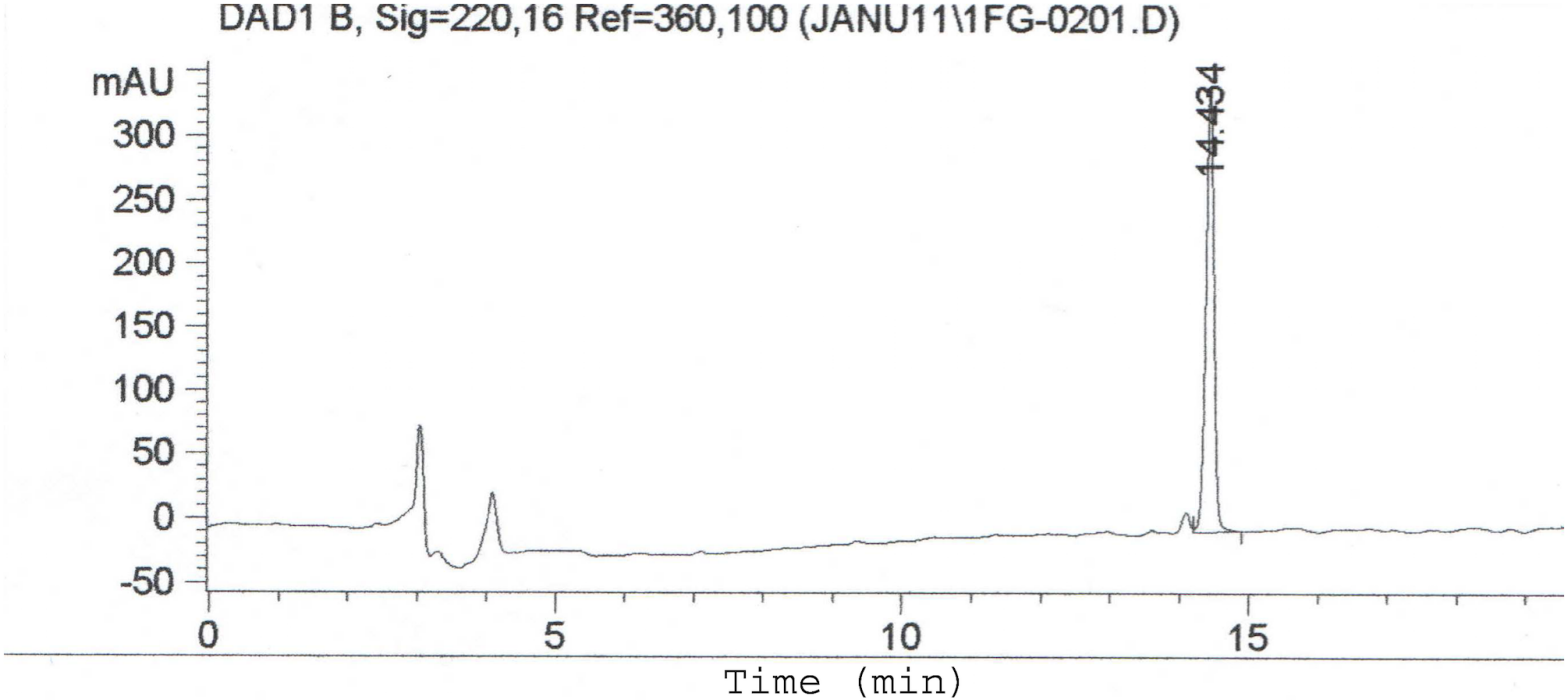
HPLC profile showing the pure compound eluted at 14.43 min.

Extracellular antibacterial compound present in the 96 h old fermented broth of *S*. *levis* was extracted with ethyl acetate. Later, it was concentrated under reduced pressure. Reddish brown colored crude thus obtained, was found to show antimicrobial activity against *K*. *pneumoniae*, *P*. *aeruginosa* and *S*. *aureus*. The compound was found to be maximum soluble in methanol suggesting its polar nature; hence it was purified with silica column, and eluted with methanol: chloroform solvent system. The purity of the fractions showing antibacterial activity was further confirmed on HPLC using reverse phase C18 column with water-acetonitrile solvent system, showing single peak with retention time of 14.434 min (Fig 3).

### Chemical characterization of purified compound

**Fig 4:**
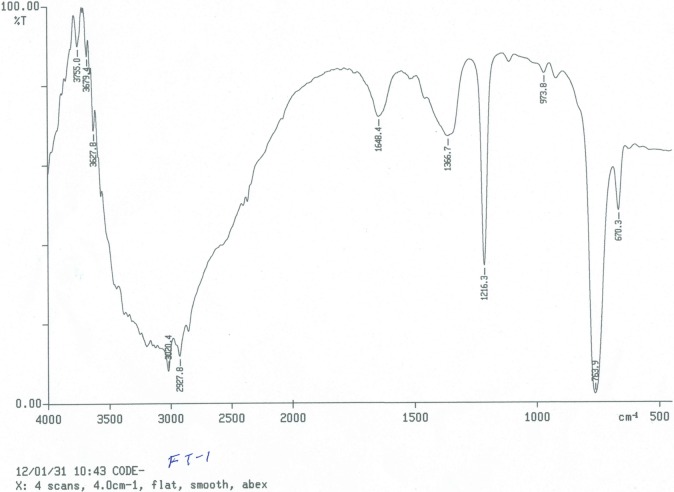
IR spectrum of the purified compound.

**Fig 5:**
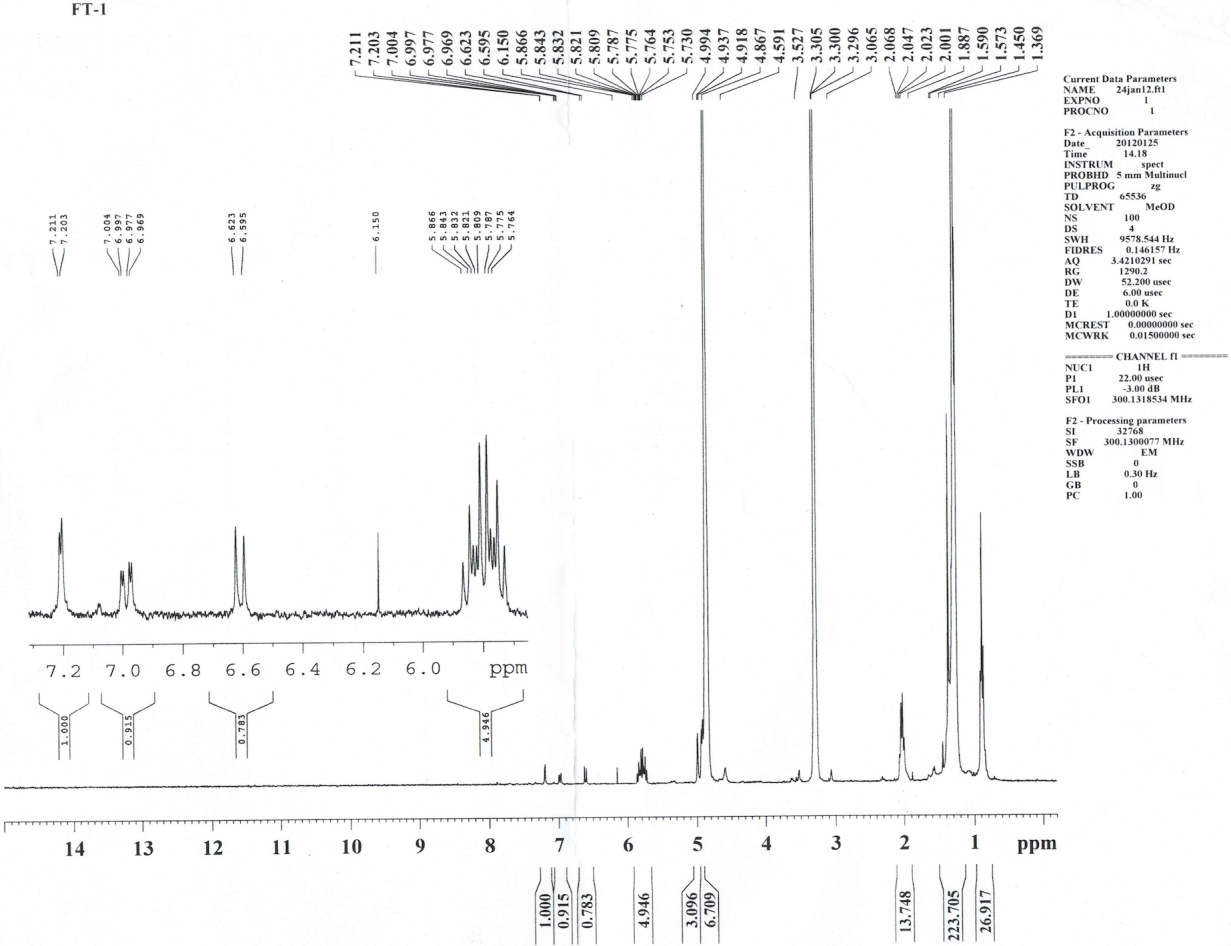
H-NMR of the purified compound.

**Fig 6:**
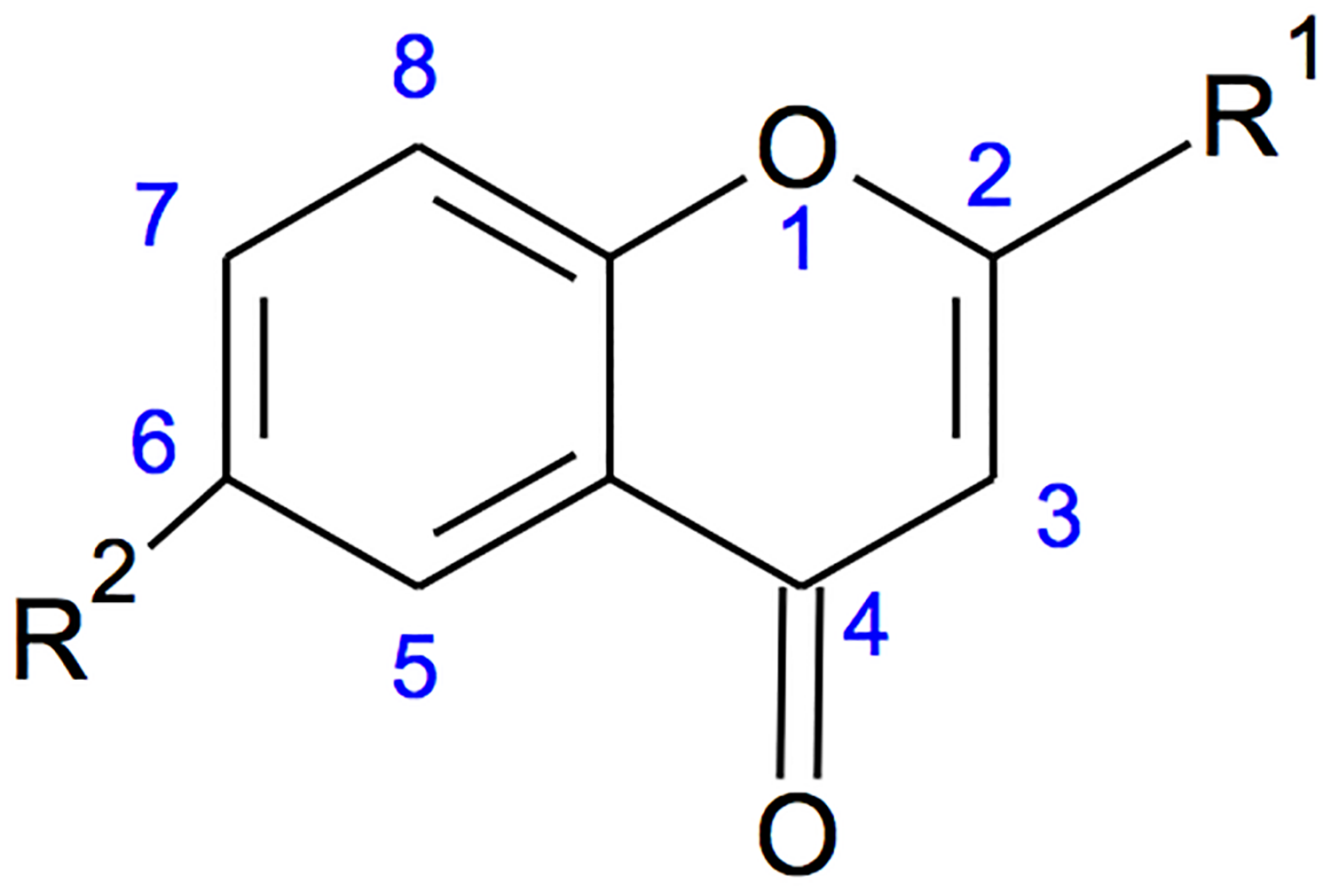
2,6-disubstituted chromone derivative.

The elemental analytical data (calculated) of the purified compound gave percentage composition of carbon, hydrogen, nitrogen, and oxygen equal to 61.31, 8.61, 2.04 and 28.02%, respectively. These results suggested a molecular formula of C_35_H_59_NO_12_. The ultraviolet (UV) absorption spectrum of the isolated compound recorded absorption bands at 240–250 and 332 nm. Absorbance within UV range confirmed the presence of unsaturation in the compound. The EI-MS/HRMS spectra of the compound exhibited m/z at 686 [M+H]^+^, 685 [M]^+^, 684, 610, 536, 347, 301 217 and 157. The presence of peak at 157 [M+H]^+^ further confirmed the presence of ‘chromone’ nucleus. IR spectrum of the compound exhibited hydroxyl and conjugated carbonyl absorption bands at 3425 and 1648 cm^-1^, respectively (Fig 4). The absorption bands at 3020 and 2927 cm^-1^ corresponded to aromatic C-H and alkyl C-H stretching, respectively, while those at 1602 and 1504 cm^-1^ attributed to C = C ring stretching. The ^1^HNMR spectrum of the compound showed signals typical of 2,6-disubstituted chromone derivative (Fig 5). Three aromatic protons appeared at δ 7.21 (1H, H-5), 6.98 (1H, H-7) and 6.61 (1H, H-8), whereas a singlet at δ 6.15 attributed to H-3 of the γ-pyrone ring. The appearance of H-5 and H-8 as doublet with ^4^*J* = 2.4 and ^3^*J* = 8.4 Hz, respectively, and H-7 as double doublet with ^3^*J* = 8.4 and ^4^*J* = 2.4 Hz clearly indicated that the ‘chromone’ ring was substituted at sixth position. In addition, the singlet signal for H-3 showed the presence of substituent at C-2 of chromone ring. The other protons of the isolated compound appeared at δ 5.76–5.86 (5H, m), 4.59–4.99 (10H, m), 3.06–3.330 (14H, m) and 1.36–2.06 (27H, m). Double bond equivalent calculation revealed that the purified antibacterial compound had seven degrees of unsaturation, which were well satisfied with the presence of ‘chromone’ skeleton and suggested that the side chains present on the 2’ and 6’ positions of chromone nucleus must be saturated in nature (Fig 6). Furthermore, the appearance of multiplet at δ 1.36–2.06 and triplet at around δ 0.9 in ^1^H NMR spectra indicated the presence of CH_2_ moieties and CH_3_ group, respectively, in the side chains.

## Discussion

In spite of the major success in the field of finding of new drug (antimicrobials) molecules, and development in their production process, infectious diseases still remain the second leading cause of death worldwide [13]. One of the main reasons of this is the development of multi-drug resistance among the pathogenic microbes, which in turn create a requirement of continuous search for new and more potential bioactive molecules. Soothingly, microbes still act as a source of novel drugs e.g., Huang et al. (2013) reported cyclic lipopeptide from *Paenibacillus ehimensis* active against *Pseudomonas aeruginosa* clinical isolates [14]. Taechowisan *et al*. (2013) reported antibacterial activity of 1-methyl ester-nigericin isolated from *Streptomyces hygroscopicus* [15]. In this series during the screening of potential microbes against *K*. *pneumoniae*, six isolates were found to possess different degrees of antimicrobial activity against *K*. *pneumoniae* MTCC 109. Out of which, the isolate *Streptomyces levis* showing significant antimicrobial activity, was found to be the most prominent strain. The initial screening of this strain has already been reported previously [16]. The results of morphological and cultural characterization of strain, suggested that the organism belonged to the *Streptomyces* sp [17]. During the literature survey it was found that the strain *S*. *levis* has been known to the biological society from long time but it was not investigated properly for metabolite production point of view. Khaliq et al. (2013) isolated *S*. *levis* along with three other *Streptomyces* strains from the soil samples from plant rhizosphere and agricultural field [18]. They examined the activity of *S*. *levis* against *Bacillus subtilis*, *Escherichia coli*, *Rhodococcus facians*, *Pseudomonas aeruginosa*, *Salmonella typhi* and *Aspergillus sp*. and reported positive activity against first four strains and negative against last two strains. Whereas, the isolated and characterized strain of *S*. *levis* reported in the present study was found to show potent activity against *Klebsiella pneumoniae* MTCC 109, *Pseudomonas aeruginosa* MTCC 741 *and Staphylococcus aureus* MTCC 96. Previously, El-Sayed et al. reported that *S*. *levis* isolated from the cultivated soil of Egypt was found to be active against some bacterial pathogen, fungal pathogens and some weeds associated with the wheat (*Triticum aestivum* L.) and maize (*Zea mays*) varieties [19].

During the structure elucidation of the active antimicrobial compound, strong absorbance at 240–250 and 332 nm suggested the presence of ‘chromone’ nucleus in the active compound [20]. Similar results were found during the characterization of the purified compound. The presence of chromone nucleus was further confirmed by the H-NMR data, which were in accord with the previously reported data of Cao and Cui, (2003) [21]. Exhaustive and exclusive search using SCI FINDER tool resulted eleven compounds having same molecular formula as our purified compound. Those compounds were oleandomycin, 2-piperidinone and derivatives of either erythromycin or tylonolide. As none of these previously reported compounds contained ‘chromone’ moiety, therefore it can be contemplated that the compound isolated in this study is not reported earlier from this strain and to the best of our knowledge is being reported for the first time from *S*. *levis*.

## Conclusion

In the present study, the soil isolate *Streptomyces levis* was found to produce a potent bioactive compound showing significant antimicrobial activity with molecular formula of C_35_H_59_NO_12_. The compound was identified as a 2,6-disubstituted chromone derivative by analyzing the spectroscopic data, and to the best of our knowledge is being reported for the first time from this strain. The present investigation also revealed the efficiency of the metabolite produced by *S*. *levis* as a good potential agent for controlling the infections against the pathogenic bacteria *K*. *pneumoniae*, *P*. *aeruginosa* and *S*. *aureus* and can be used as a promising antimicrobial drug in the near future.

## References

[1] Fung CP, Chang FY, Lee SC, Hu BS, Kuo BI, Liu CY, et al (2002) A global emerging disease of *Klebsiella pneumoniae* liver abscess: is serotype K1 an important factor for complicated endophthalmitis? Gut. 50:420–424 11839725 10.1136/gut.50.3.420 PMC1773126

[2] Bodey GP, Bolivar R, Fainstein V, Jadeja L (1983) Infections caused by *Pseudomonas aeruginosa*. Rev Infectious Diseases. 5:279–313 6405475 10.1093/clinids/5.2.279

[3] El Nekidy WS, Mooty MY, Attallah N, Cardona L, Bonilla MF, Ghazi IM. (2017) Successful treatment of multidrug resistant *Klebsiella pneumoniae* using dual carbapenem regimen in immunocompromised patient. IDCases. 9:53–55. doi: 10.1016/j.idcr.2017.06.005 28660131 10.1016/j.idcr.2017.06.005 PMC5480227

[4] Harvey I, Cormier Y, Beaulieu C, Akimov VN, Mériaux A, Duchaine C. (2001) Random amplified ribosomal DNA restriction analysis for rapid identification of thermophilic actinomycete-like bacteria involved in hypersensitivity pneumonitis. Syst Appl Microbiol. 24:277–284 doi: 10.1078/0723-2020-00034 11518332 10.1078/0723-2020-00034

[5] Tripathi C, Praveen V, Singh V, Bihari V. (2004) Production of antibacterial and antifungal metabolites by *Streptomyces violaceusniger* and media optimization studies for the maximum metabolite production. Med Chem Res. 13:790–799.

[6] Singh N, Rai V, Tripathi C. (2012) Production and optimization of oxytetracycline by a new isolate *Streptomyces rimosus* using response surface methodology. Med. Chem. Res. 21:3140–3145.

[7] Vining LC. (1990) Functions of secondary metabolites. Annual Rev Microbiol. 44:395–427. 2252388 10.1146/annurev.mi.44.100190.002143

[8] Gogoi N, Yadav R, Dutta P, Bordoloi G. (2005) Studies on antimicrobial activity of actinomycete strains isolated from Majuli, a river island. Ind J Microbiol. 45:231

[9] Sharma M. (2014) Actinomycetes: Source, identification, and their applications. Int J Curr Microbiol App Sci. 3:801–832.

[10] Singh V, Tripathi C, Bihari V. (2008). Production, optimization and purification of an antifungal compound from *Streptomyces capoamus* MTCC 8123. Med Chem Res. 17:94–102.

[11] Holt JG, Krieg NR, Sneath PH. Bergey’s manual of determinative bacteriology. (1994)

[12] National Committee for Clinical Laboratory Standards. Approved standard M7-A5 Methods for dilution antimicrobial susceptibility tests for bacteria that grow aerobically. 4^th^ edn (Wayne, PA: NCCLS, 2000.

[13] de Lima Procópio RE, da Silva IR, Martins MK, de Azevedo JL, de Araújo JM. (2012) Antibiotics produced by *Streptomyces*. Braz J Infect. Dis. 16:466–471. doi: 10.1016/j.bjid.2012.08.014 22975171 10.1016/j.bjid.2012.08.014

[14] Huang Z, Hu Y, Shou L, Song M. (2013) Isolation and partial characterization of cyclic lipopeptide antibiotics produced by *Paenibacillus ehimensis* B7. BMC Microbiol.13:87 doi: 10.1186/1471-2180-13-87 23594351 10.1186/1471-2180-13-87 PMC3637185

[15] Taechowisan T, Chanaphat S, Ruensamran W, Phutdhawong WS. (2013) Antibacterial activity of 1-methyl ester-nigericin from Streptomyces hygroscopicus BRM10; an endophyte in Alpinia galanga. J Appl Pharmaceut Sci. 3:104–109.

[16] Singh V, Haque S, Singh H, Verma J, Vibha K, Singh R, et al (2016) Isolation, Screening, and Identification of Novel Isolates of Actinomycetes from India for Antimicrobial Applications. Front Microbiol. 7:1921 doi: 10.3389/fmicb.2016.01921 27999566 10.3389/fmicb.2016.01921 PMC5138215

[17] Watve MG, Tickoo R, Jog MM, Bhole BD. (2001) How many antibiotics are produced by the genus *Streptomyces*? Arch Microbiol. 176:386–390. doi: 10.1007/s002030100345 11702082 10.1007/s002030100345

[18] Khaliq S, Ghauri MA, Akhtar K. (2013) Isolation, identification and optimization of fermentation parameters for improved production of antimicrobial compounds from indigenous *Streptomyces* isolates. Afr J Microbiol Res. 7:1874–1886.

[19] El-Sayed MH, El-Aziz ZKA, Abouzaid AM. (2014) Efficacy of extracellular metabolite produced by *Streptomyces levis* strain LX-65 as a potential herbicidal agent. J Ame Sci. 10(11):169–180.

[20] Griffiths P, Ellis G. (1972) Benzopyrones—VI: The ultraviolet absorption spectra of chromone and 2-substituted chromones Spectrochimica Acta Part A: Mol Spectroscopy. 28:707–713.

[21] Cao LH, Cui PY. (2003) Synthesis of 2‐Dihydrooxadiazolinyl Chromones. J Chinese Chem Soc. 50:903–908.

